# Supplementary figures and images for: New alignment-based sequence extraction software (ALiBaSeq) and its utility for deep level phylogenetics
Source: PeerJ. 2021 Mar 31;9:e11019. doi: 10.7717/peerj.11019 (PMC8019319; doi:10.7717/peerj.11019)

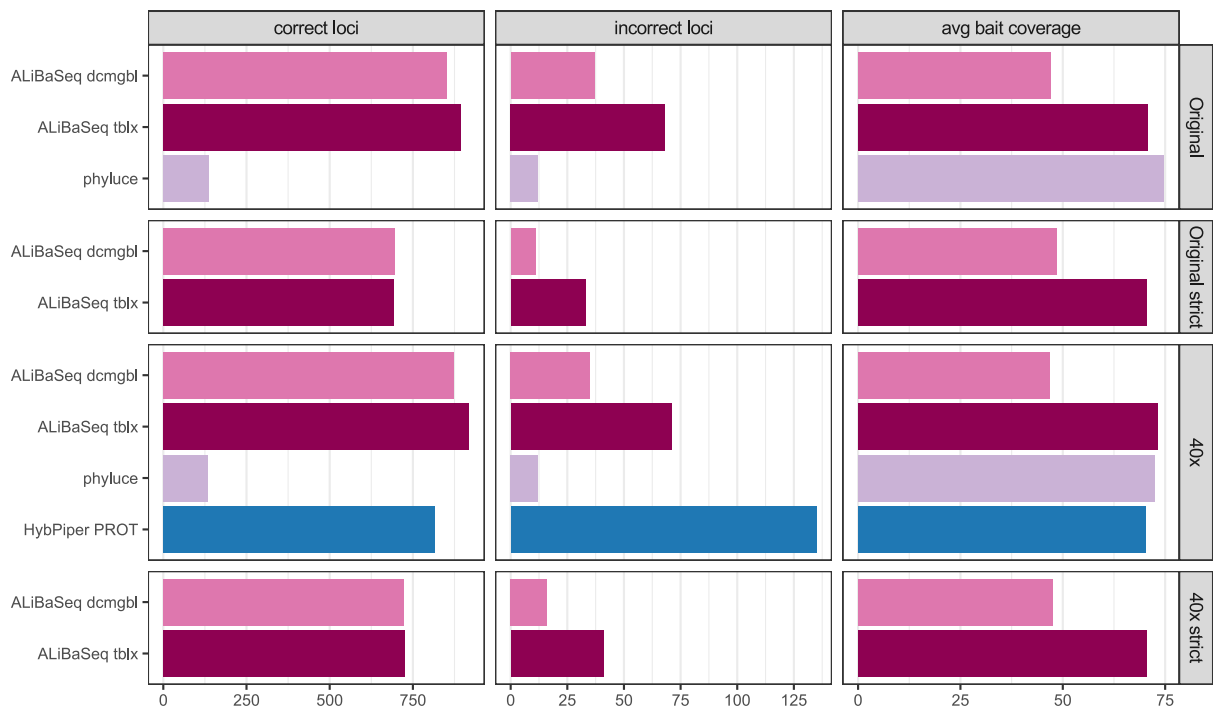

Supplement: Supplemental Information 2 — The correct loci panel shows amount of loci with a high sequence identity to the A. trichopoda bait region sequence, the incorrect loci panel shows amount of loci with a low sequence identity to the A. trichopoda bait region sequence, the avg bait coverage panel shows the average percentage of the bait region recovered. Vertical panels refer to different datasets and RBH check modes: original – original assembly with relaxed RBH check (ALiBaSeq only), original strict – original assembly with strict RBH check, 40x – simulated reads with 40x depth and relaxed RBH check (ALiBaSeq only), 40x strict – simulated reads with 40x depth and strict RBH check. Abbreviations are as follows: dcmgbl – discontinuous megablast, tblx – tblastx. [file peerj-09-11019-s002.pdf]
